# Unveiling the shield: decoding UPSIDE^®^-induced biochemical responses against *Botrytis cinerea* in grapevine

**DOI:** 10.3389/fpls.2025.1674937

**Published:** 2025-11-05

**Authors:** Giulia Scimone, Lorenzo Mariotti, Zuzana Gelová, Lisa Milanollo, Claudia Pisuttu, Elisa Pellegrini, Cristina Nali

**Affiliations:** ^1^ Department of Agriculture, Food and Environment, University of Pisa, Pisa, Italy; ^2^ Kwizda Agro GmbH, Vienna, Austria

**Keywords:** crop protection, grey mold, phytohormones, priming, resistance elicitors, IR, signaling molecules, *Vitis vinifera*

## Abstract

*Botrytis cinerea* Pers. is one of the major threats to grapevine, causing substantial losses in both yield and fruit quality. In light of the evolving regulatory frameworks and growing concerns over the use of chemical fungicides, the search for new green and affordable tools has become increasingly relevant. This study aims to investigate biochemical changes on grapevine induced by the application of UPSIDE^®^ (U), a new yeast-based product developed for organic viticulture. Foliar spray applications of U were carried out once a week for three consecutive weeks on potted grapevines (U^+^), while other plants were sprayed with sterile water (U^-^). At the end of the treatments, half plants from each group were *B. cinerea* (Bc^+^)- or mock-inoculated (Bc^-^). Fully expanded leaves were sampled at 24 hours after each spray, and at 0-, 1-, 3-, 24- and 48-hours post inoculation (hpi), and then used for biochemical analysis and microscopic observations. In U^+^/Bc^-^ plants, hydrogen peroxide levels increased at all analysis times (+50% on average), along with a 3-fold rise in ethylene/jasmonic acid (Et/JA) ratio at 3 hpi, in comparison to U^-^/Bc^-^. In U^-^/Bc^+^ plants, the Et/JA ratio remained high throughout the experiment, peaking at 48 hpi, while salicylic acid (SA) dropped at 1 hpi (more than 7-fold and –42%, respectively, compared to U^-^/Bc^-^ ones). Similarly, U^+^/Bc^+^ plants showed increased levels of Et/JA ratio from 3 to 48 hpi and abscisic acid at 3 hpi (5- and 16-fold, respectively, compared to U^-^/Bc^-^), with a SA decrease at 1 hpi (–63%, in comparison to U^-^/Bc^-^). Microscopic observations revealed fully hyphal spread on U^-^/Bc^+^ leaves, while U^+^/Bc^+^ showed only slightly formed and misshaped germ tubes. Results confirmed the potential of U in triggering plant defense responses, thus protecting grapevine leaves against Bc.

## 1 Introduction

Plant diseases have posed significant threats to farmers and stakeholders within all agricultural systems (O’Brien, 2017). Indeed, losses due to plant pathogens are not only referred as reduced yields, but they could be expanded in terms of low quality of crops and their products and high production costs (Reddy et al., 2024). The agricultural practices conventionally used for disease management take advantage of various approaches, including (i) plant breeding and genetic engineering for the development of tolerant/resistant genotypes, (ii) application of physical methods (exploiting heat treatments, UV radiation, modified/controlled atmosphere; Pisuttu et al., 2023; Santin et al., 2025), and (iii) agrochemicals distribution (which remains the most widely used strategy; Thambugala et al., 2020). In recent years, several chemical fungicides have been restrained due to the increasing concerns about environmental sustainability (i.e., their persistence and/or accumulation in the environment), increased acquired resistance in fungal strains and human health (McLaughlin et al., 2024). Therefore, there is an urgent need to replace unsustainable practices in agriculture, with growing interest in the biological control strategies.

Biological control (hereafter referred to as “biocontrol”) is a plant disease management strategy that involves the use of beneficial microorganisms and/or their metabolites, to suppress pathogens, boost plant defenses, and alter environmental conditions to reduce disease impact (He et al., 2021). Biocontrol approaches can be used alone or combined with an integrated strategy (Collinge et al., 2022). According to their modes of action, biocontrol agents (BCAs) can be classified into three categories: (i) pathogens suppressors, which develop a direct antagonism for space and nutrients competition, antibiosis or parasitism; (ii) defense activators, which enhance the plant’s own defense mechanisms; and (iii) ecological regulators, which modify the environment to favor natural enemies or competitors of pathogens (Raymaekers et al., 2020; Palmieri et al., 2022). The exogenous application of molecules, which could be either chemicals, plant or non-pathogenic and microbe extracts (such as filamentous fungi, bacteria, and yeasts), can activate internal plant alerting mechanisms. This occurs due to a specific plant phenotype named “induced resistance” (IR), which involves a decreased susceptibility following stimulation by external factors such as pathogens, insect herbivores, wounding, beneficial microbes, or chemical agents (De Kesel et al., 2021). Moreover, the IR phenotype includes both direct induction of defense responses (activated immediately upon contact with the IR stimulus) and primed defense responses (where defenses are triggered more quickly, strongly, or efficiently upon later exposure to a threat) (Mauch-Mani et al., 2017). A first general attempt in response to external stress factors involves the production of reactive oxygen species (ROS), with a special regard for hydrogen peroxide (H_2_O_2_), which usually contributes to the activation of a more sophisticated molecules crosstalk based upon pathogen nature recognition (Smirnoff and Arnaud, 2019; Shu et al., 2024). Thus, a second alerting defense layer, mostly represented by signaling molecules (principally phytohormones) such as ethylene (Et), salicylic, jasmonic and abscisic (SA, JA, and ABA) acids, is crucial for triggering pathogen-specific defense mechanisms (AbuQamar et al., 2017). In these terms, IR-elicitation represents an evolutive advantage in terms of energy investment to counteract infection processes (van Hulten et al., 2006). Consequently, IR induction could be considered a resilient strategy to exploit in the matter of crop protection and disease management (Urban et al., 2022).

An economically important trade in terms of plant yields is represented by the grapevine (*Vitis vinifera* L.) sector due to its extended cultivation for wine, distilled liquors, juice, table grapes and raisin production (FAO-OIV, 2016; Gambetta et al., 2020). The growth and productivity of *V. vinifera* varieties could be severely affected by many threats endangering both pre- and post-harvest. To cope with them and meet the quali-quantitative production standards, an intensive agrochemicals schedule is often required, leading winegrowers to frequently apply long-term fungicides (Provost and Pedneault, 2016; Pertot et al., 2017; Brulé et al., 2024). Among the most significant threats, *Botrytis cinerea* Pers. (Bc), a common polyphagous pathogen which represents the causal agent of grey mold, is well-known by winegrowers for its undesirable effects on grape berries (Toffolatti et al., 2020). *Botrytis cinerea* represents the cause of 20-50% yield grape loss in global viticulture (Fedorina et al., 2022) and, together with quantity, also berries quality is compromised due to the pathogen presence (Pertot et al., 2017). Even if the impactful effects are mostly recorded on fruits, other necrotic tissues, such as leaves and flowers, have been identified as potential inoculum sources (Fedele et al., 2020). Indeed, it is well established that if both humidity and temperatures are high during the vegetative season, the pathogen can prosper in the vineyard, and thus be preserved in soil, leaves and/or flowers (Toffolatti et al., 2020; Rahman et al., 2024). Consequently, active prevention of this pathogen may act as an important player in the grey mold-grapevine pathosystem to reduce the consequences of infection (Calvo-Garrido et al., 2014). Therefore, Bc control in vineyards usually involves the routine application of chemical fungicides at key growth stages (i.e., flowering, pre-bunch closure, veraison and before harvest; Altieri et al., 2024), combined with varying degrees of canopy management (i.e., bunch trash removal, leaf removal and mechanical thinning; Mundy et al., 2022). However, this approach overlooks the actual infection risk present in the field and contributes to the development of resistance to botryticides in Bc (González‐Domínguez et al., 2019). For this reason, Bc is considered a ‘high-risk’ pathogen for fungicide resistance development (Walker et al., 2017). Consequenlty, the existence of multi-resistant strains (Hahn, 2014), combined with the clear inefficiency of the major part of available chemicals (Jelenić et al., 2024) and the growing regulatory constraints in matter of plant protection products (Carisse and Heyden, 2025), is driving viticulture for request of new green and affordable tool to use against this pathogen as well.

Although progress has been made in biological control, the effectiveness of yeast-based formulations as resistance inducers in grapevine remains poorly understood. Therefore, the aim of this work was to study the potential of a new *Saccharomyces cerevisiae* Meyen extract formulate named “UPSIDE^®^” (U), developed by Kwizda Agro GmbH (ABE-IT 56, *S. cerevisiae* Stamm DDSF623, Kwizda Agro GmbH, Vienna, Austria; Scimone et al., 2024) as IR-inducer in *V. vinifera*-Bc pathosystem, by answering to the following questions: (i) Does U trigger biochemical defense responses in uninoculated grapevine leaves?, (ii) Does the signal similarly occur also in inoculated ones?, and (iii) Does U have any fungistatic/fungicidal effects on Bc?

## 2 Materials and methods

To determine the optimal *in vivo* application of U, bioassays were performed to assess its potential phytotoxic effects by evaluating leaf damage across increasing concentrations, while the U- direct antifungal activity against Bc mycelial growth was investigated by using the amended potato dextrose agar (PDA) method.

### 2.1 Leaf discs phytotoxicity assay

The potential phytotoxicity of U was visually evaluated on leaf discs following the method described by La Torre et al. (2014), with modifications. Forty leaf discs (Ø 2 cm) were excised from untreated leaves of *V. vinifera* cv. Sangiovese (clone F9-A5-48; rootstock 110 Richter) by using a cork borer. The discs were then surface sterilized with 0.5% sodium hypochlorite (v/v in water), followed by spraying with U solutions diluted in water at varying concentrations: 0.25 (suggested concentration), 0.5 and 1% (v/v). The suggested concentration was previously selected according to preliminary tests (see also Scimone et al., 2024). The double and quadruple concentrations were selected according to EPPO guidelines (EPPO, 2014). Leaf discs sprayed with only water were used as a control. After 24 h, the discs were observed under a stereomicroscope (Leica S9i, Leica, Wetzlar, Germany) to assess the presence of visible injuries related to phytotoxic effects. Ten leaf discs were used for each treatment.

### 2.2 *In vitro* evaluation of the antifungal activity of UPSIDE^®^ on mycelium growth

A fungal strain of Bc (8335) stored in the fungal collection of the Department of Agriculture, Food and Environment (DAFE, University of Pisa) was grown on PDA (42 g L^-1^; BioLife, Milan, Italy) added with streptomycin sulfate (0.1 g L^-1^) in Petri dishes (Ø 9 cm) and incubated for 7 days at 23 °C and 12/12 h photoperiod. According to Scimone et al. (2025), the formulate was added to sterile PDA during solidification at the concentration of 2.5 mL L^-1^ (v/v), as suggested by the company. The microbiological medium, amended with sterile water at the same concentration, was used as control. Petri dishes (Ø 6 cm), containing the amended or control medium, were inoculated with a plug (Ø 0.6 cm) of an actively growing colony of Bc and measured daily until the control colony reached the edges. Ten independent replicates were performed for each treatment.

### 2.3 Plant material, inoculation with *Botrytis cinerea* and experimental conditions

**Figure 1:**
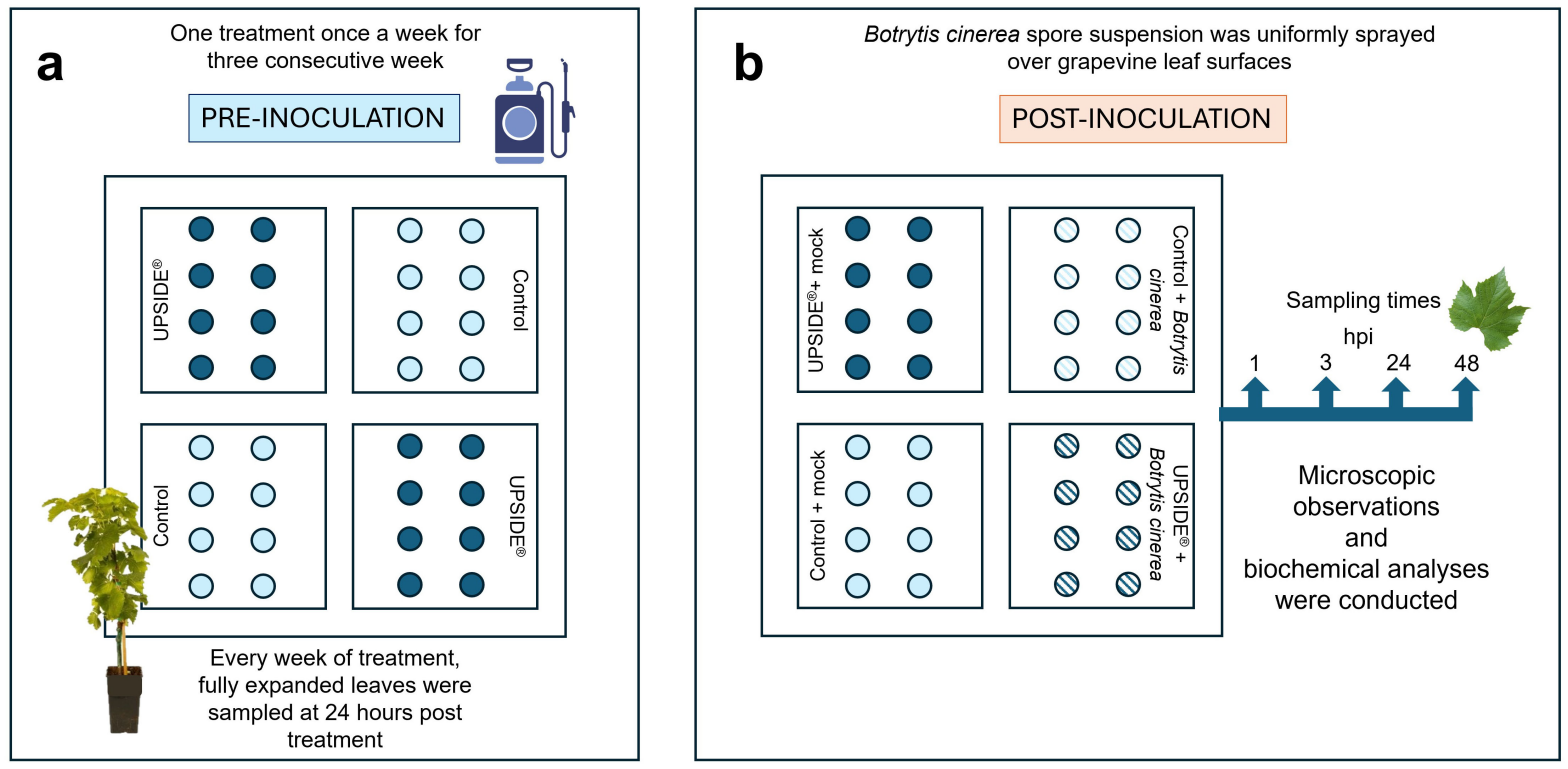
Schematization of experimental design. **(a)** Pre-inoculation: leaves of 32 plants of *Vitis vinifera* cv. Sangiovese were sprayed until runoff once a week for three consecutive weeks with sterile water (control; light blue circle) or UPSIDE^®^ (blue circle). **(b)** Post-inoculation: after the pre-inoculation time frame, plants were mock- (control + mock, light blue circle; UPSIDE^®^ + mock, blue circle) or *Botrytis cinerea*-inoculated (control + *B. cinerea*, filled light blue circle; UPSIDE^®^ + B. cinerea, filled blue circle). hpi, hours post inoculation.

In July 2022, 32 four-year-old plants of *V. vinifera* cv. Sangiovese, purchased from a local nursery, were placed in a greenhouse at San Piero a Grado (Pisa, Italy), which is owned by the DAFE. They were acclimated for 2 weeks under controlled conditions (temperature of 25±5 °C, relative humidity of 80±10% and photon flux density 530 µmol m^-2^ s^-1^ at plant height provided by incandescent lamps with light/darkness 12/12 h photoperiod) and kept well-watered. Temperature and RH were selected according to the optimum for Bc development, with minor modifications due to the plant needs and the phytotron used for the experiment (Fedele et al., 2020; Li et al., 2023). Subsequently, plants were divided into two groups: 16 grapevines were foliar sprayed with U (U^+^; 2.5 mL L^-1^, v/v in water, Scimone et al., 2024), once a week for three consecutive weeks, while the remaining 16 plants were sprayed with sterile water as control (U^-^). Fully expanded leaves from the two groups were sampled 24 h after each foliar application, to monitor the immediate plant response (24 h after the treatment resulted the time at which plants appeared more responsive at both biochemical and molecular level; Scimone et al., 2024). At the end of the treatments, eight plants of each group were transferred into plexiglass boxes (60×60×110 cm) under the same growth chamber conditions to proceed with the inoculation. For this purpose, a flask (0.5 L) of Bc liquid culture was obtained scratching conidia in a sucrose-yeast extract solution (2% and 0.05%, w/v in water, respectively), and maintained in an orbital homogenizer (711 CT, Asal, Milan, Italy) set at 150 rpm for 48 h at room temperature. The conidial concentration was measured through a hemocytometer (Henneberg-Sander, Giessen Lützellinden, Germany) and adjusted to 10^5^ conidia mL^-1^. A sterile solution was used as control (mock inoculation). After the application by brush of carborundum powder to facilitate the infection, 5 mL of Bc spore suspension per plant was uniformly sprayed on *V. vinifera* leaf surfaces (Bc^+^). The remaining plants were brush-treated with the same carborundum powder and mock inoculated with sterile solution (Bc^-^). Thus, the 4 sets were named as follows: U^-^/Bc^-^ (plants treated with sterile water and mock inoculated), U^+^/Bc^-^ (plants treated with U and mock inoculated), U^-^/Bc^+^ (plants treated with sterile water and inoculated with Bc), and U^+^/Bc^+^ (plants treated with U and inoculated with Bc). Fully expanded leaves were harvested from five plants of each group at 0-, 1-, 3-, 24- and 48-hours post inoculation (hpi). At each time point, part of the sample was immediately used for microscopic observations and Et determination, while the remaining material was ground in liquid nitrogen and kept at -80°C until further biochemical analyses (**Figure 1**).

### 2.4 Oxidative stress markers

Hydrogen peroxide levels were quantified using the AmplexTM Red Hydrogen Peroxide/Peroxidase Assay Kit (Molecular Probes, Life Technologies Corp., Carlsbad, CA, USA), following the protocol described by Pisuttu et al. (2020). Frozen samples (50 mg) were extracted in 1 mL of 20 mM potassium-phosphate buffer (pH 6.5), incubated for 30 min at 25 °C in the dark. Then, H_2_O_2_ was determined by using a Victor3–1420 Multilabel Couter microplate reader (Perkin Elmer Inc., Waltham, MA, USA) at 530 and 590 nm for the excitation and emission of resorufin fluorescence, respectively, and plotted against a standard curve ranging from 0 to 20 μM H_2_O_2_.

Oxidative damage was assessed in terms of lipid peroxidation by performing the thiobarbituric acid-reactive-substances (TBARS) assay and checking for malondialdehyde (MDA) accumulation, as reported by Hodges et al. (1999), with minor modifications. Frozen samples (100 mg) were homogenized in 1 mL of 0.1% trichloroacetic acid (TCA; w/v, in water). A 200 µL sample aliquot was added to: i) 300 µL of 0.1% TCA (w/v, in water) and 500 ml of 20% TCA (w/v, in water); ii) 300 µl of 0.1% TCA (w/v, in water) and 500 µl of 0.5% thiobarbituric acid (w/v, in 20% TCA). Samples were then incubated for 30 min at 95 °C, then centrifuged at 10,000*g* for 10 min. The determination was performed with the same microplate reader previously reported at 532 nm and corrected for non-specific turbidity by the subtraction of 440 and 600 nm absorbance.

For each parameter, five biological replicates, technically repeated three times, were used.

### 2.5 Phytohormones

Ethylene emission was determined following the method described in Scimone et al. (2024). Fresh whole leaves (250 mg) were closed in 15 mL glass vials and, after a 2 h incubation, one mL of gas, taken from the headspace of each sample through a hypodermic syringe, was injected into an Agilent 8890B gas chromatograph equipped with an Agilent HP-PLOT/Q+PT capillary column (30 m × 0.32 mm; coating thickness 0.20 μm), and an Agilent 5977B single quadrupole mass detector (Agilent Technologies Inc., Santa Clara, CA, USA).

Jasmonic acid was determined according to the protocol of Modesti et al. (2023). Frozen samples (100 mg) were extracted with 1 mL of pure methanol (MeOH). After 30 min centrifugation at 13,000*g*, resulting supernatants were filtered and evaporated at 35 °C under a vacuum (RVC 2–25 CDplus, Martin Christ Gefriertrocknungsanlagen GmbH, Osterode, Germany). The residues were re-suspended with 750 μL of ethyl acetate and injected into a GC-MS (previously described) equipped with an Agilent DB-5MS (UI) capillary column (30 m × 0.25 mm; coating thickness 0.25 μm).

Free and conjugated SA were determined following the method described in Scimone et al. (2024). Frozen samples (200 mg) were extracted with 1 mL of 90% MeOH (v/v, in water). After 15 min centrifugation at 10,000*g*, resulting supernatants were transferred, and the pellet was re-extracted in 0.5 mL of pure MeOH. Supernatants from both extractions were combined and left evaporated under vacuum at 35 °C. The residue was resuspended in 0.25 mL of 5% (w/v) TCA and partitioned twice using 0.8 mL of a 1:1 (v/v) mixture of ethyl acetate/cyclohexane. The upper phase containing free SA was concentrated under vacuum at 35 °C, while the lower aqueous phase with conjugated SA was hydrolysed by adding 0.3 mL of 8 N HCl and incubating at 80 °C for 1 h. The SA collected from each upper and lower phase was separately dissolved in 500 µL of 0.2 M sodium acetate buffer (pH 5.5), water (90%) and MeOH (10%). Separations were performed by an ultra-high pressure liquid chromatography (UHPLC) Dionex UltiMate 3000 system equipped with an Acclaim 120 C18 column (5 μm particle size, 4.6 mm internal diameter × 150 mm length; Thermo Scientific, Waltham, MA, USA), maintained at 40 °C, and a UltiMate™ 3000 Fluorescence Detector (Thermo Scientific, Waltham, MA, USA) with excitation at 305 nm and emission at 407 nm. The SA was eluted using 100% solvent (sodium acetate/MeOH, 90:10, v/v) for 20 min. The flow rate was 0.8 mL min^-1^.

Abscisic acid was determined using the Phytodetek^®^ Immunoassay Kit for ABA (Agdia Elkhart, IN, USA), according to Pisuttu et al. (2023). Frozen samples (100 mg) were extracted with 1 mL of distilled water. After 30 min centrifugation at 13,000*g*, resulting supernatant was diluted 10 times. The determination of ABA was performed at 415 nm by using the same microplate reader, previously reported.

For Et determination, five biological replicates were used. Concerning all other analyses, five biological replicates, technically repeated three times, were used.

### 2.6 Microscopic observations

*Botrytis cinerea* hyphal structures developed in *V. vinifera* leaves were stained according to Modesti et al. (2023), to investigate the surface infection process after the inoculation. Tissue cuttings (1-cm) obtained through sampled leaves were boiled for 1.5 min in a mixture of 95% ethanol (v/v, in water):lactophenol cotton blue (2:1, v/v). After 48 h, samples were washed with distilled water and kept for 30 min in a chloral hydrate:water solution (2:1). Stained tissues were then fixed on glasses slides with 50% glycerol and visualized using an optical microscope (DM 4000^®^ B led, Leica, Wetzlar, Germany). Photomicrographs were taken with a Canon PowerShot S50^®^ camera (Canon Italia, Milan, Italy). For each treatment, a total of 20 sections were produced (four pieces for leaves, using five independent biological replicates).

### 2.7 Statistical analysis

Normal distribution of data was first assessed by the Shapiro-Wilk test. The effect of ‘treatment’ was investigated using a Student’s *t*-test for the data obtained at plant level at pre-inoculation stage. A two-way analysis of variance (ANOVA) was used to evaluate the effect of ‘treatment’, ‘time’ and their interaction on Bc mycelium growth, and at post-inoculation stage; Tukey’s HSD *post hoc* test was used for mean comparisons. A canonical discriminant analysis (CDA) was performed to evaluate the overall effect and differences of the hormones analyzed (i.e., Et/JA, SA, ABA and H_2_O_2_) among the experimental groups at post-inoculation stage. Then, Pearson’s correlation coefficient was assessed to identify which variables significantly contributed to group separation. Pearson’s correlation coefficient was calculated between individual variables and the scores of the first and second canonical functions, which explained 55.9 and 22.9% of the total variance, respectively. Differences were considered statistically significant at *P* ≤ 0.05. Statistical analyses were performed in JMP^®^ Pro 14.0 (SAS Institute Inc., Cary, NC, USA) and SigmaPlot 12.5 (Sigmaplot. Ink, Systat Software Inc.).

## 3 Results

### 3.1 Phytotoxicity visual assessment

**Figure 2:**
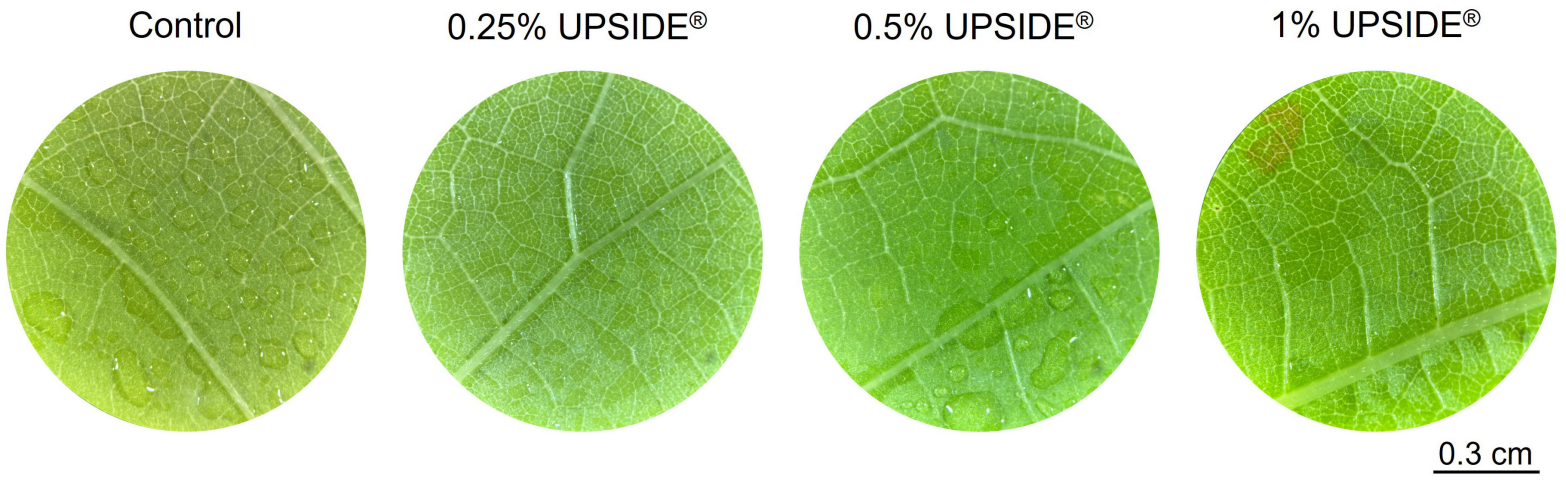
Visual assessment of phytotoxic effects on *Vitis vinifera* cv. Sangiovese leaf discs sprayed with water (Control) and UPSIDE^®^ at 0.25, 0.5 and 1% (v/v in water).

Microscopic observations provided a first assessment of the direct U-effect on *V. vinifera* cv. Sangiovese plants. Grapevine leaves treated with the recommended concentration (0.25%) and double of the recommended concentration (0.5%) showed no phytotoxic effects in comparison to the controls. Conversely, necrotic lesions were observed on both leaf surfaces following the application of the highest U concentration (1%; **Figure 2**).

### 3.2 UPSIDE^®^ *in vitro* antifungal activity

**Figure 3:**
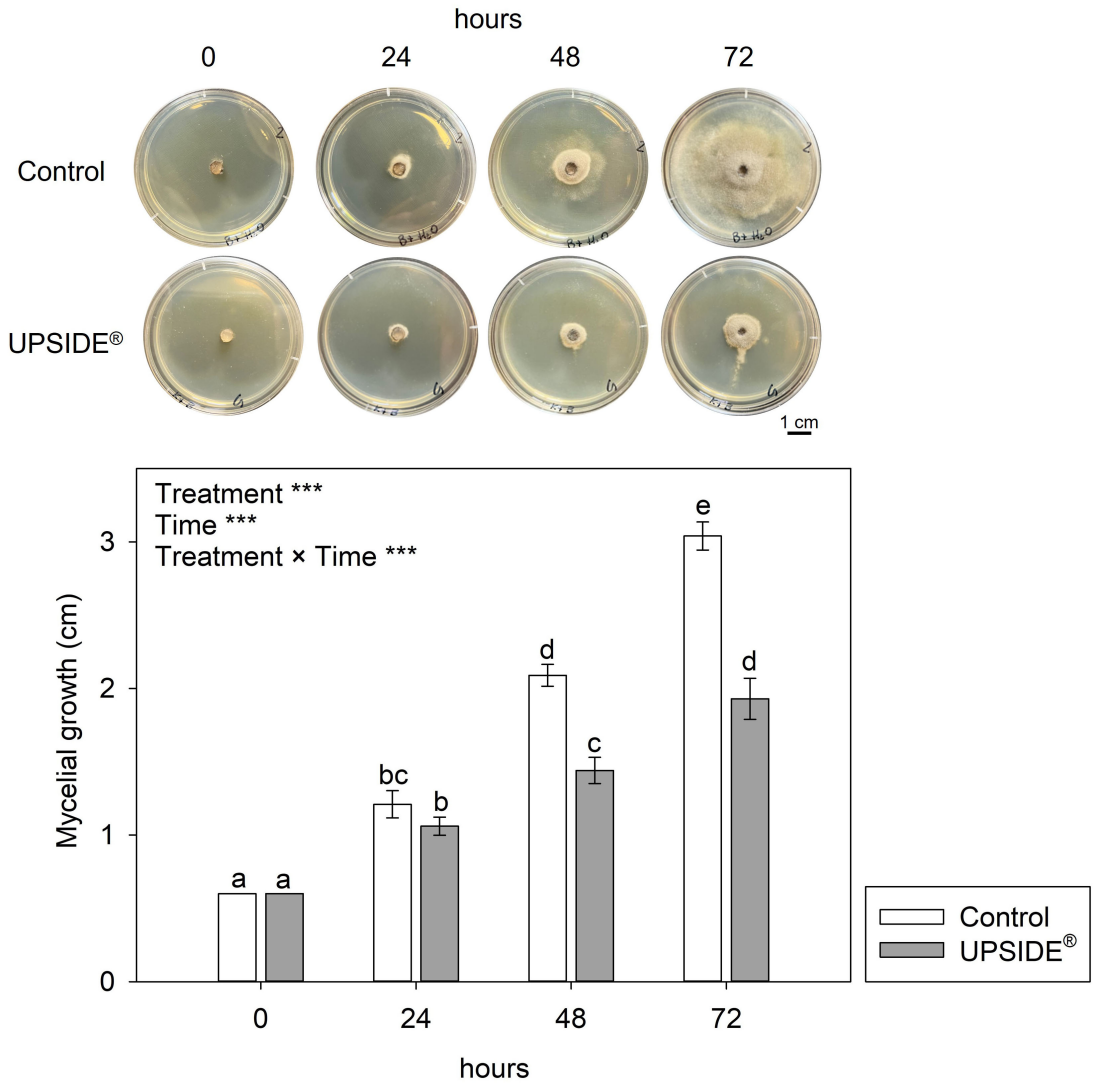
Mycelial growth of *Botrytis cinerea* in Potato Dextrose Agar added with water (Control; white bar) and UPSIDE^®^ (grey bar). Data are shown as mean±standard deviation (n = 10). The results of a two-way analysis of variance (with ‘treatment’ and ‘time’ as variability factors) have been reported (***P ≤ 0.001). Different letters indicate significant differences among means according to Tukey’s HSD *post hoc* test.

The two-way ANOVA showed that the effects of ‘treatment’, ‘time’ and their interaction were significant in evaluating the antifungal activity of U on Bc mycelial growth (**Figure 3**). Notably, U-amended PDA caused strong inhibition of fungal growth at both 48 and 72 h (–31 and –36%, respectively, in comparison to the control). No significant differences were observed at 24 h.

### 3.3 Levels of oxidative stress markers and hormones at pre-inoculation stage

At 24 h after the first weekly treatment U^+^-treated leaves showed higher values of H_2_O_2_ and MDA than U^-^ ones (+50 and +15%, respectively; **Table 1**). No significant differences were observed in relation to Et/JA ratio, SA and ABA contents. At 24 h after the second weekly treatment, U^+^ leaves recorded higher concentrations of H_2_O_2_ (+86%), and lower levels of both Et/JA ratio and SA (–46 and –49% in comparison to U^-^ ones). No significant differences were observed regarding the remaining compounds (i.e. MDA and ABA). At 24 h after the third weekly treatment, only H_2_O_2_ values were recorded to significantly increase (+46%, in comparison to U^-^ ones).

**Table 1:** Weekly variation in hydrogen peroxide (H_2_O_2_), malondialdehyde (MDA), ethylene (Et) and jasmonic acid (JA) ratio, salicylic acid (SA) and abscisic acid (ABA) contents (expressed as fresh weight, FW) in *Vitis vinifera* cv.

	1^st^ week of treatment			2^nd^ week of treatment			3^rd^ week of treatment		
Parameter	U^-^	U^+^	*P*	U^-^	U^+^	*P*	U^-^	U^+^	*P*
H_2_O_2_ (nmol g^-1^ FW)	120±5	180±3	***	117±2	217±1	***	106±3	135±3	**
MDA (nmol g^-1^ FW)	20±1	23±1	**	24±1	25±3	ns	22±2	28±1	ns
Et/JA (nl µg^-1^ h^-1^ FW)	0.22±0.01	0.17±0.02	ns	0.15±0.01	0.08±0.01	***	0.14±0.01	0.13±0.01	ns
SA (µg g^-1^ FW)	3.5±0.4	3.9±0.1	ns	4.9±0.2	2.5±0.2	***	1.8±0.1	1.7±0.2	ns
ABA (ng g^-1^ FW)	256±57	203±42	ns	158±12	150±19	ns	200±22	144±17	ns

Sangiovese leaves treated with water (control, U^-^) and UPSIDE^®^ (U^+^) throughout the whole period of the experiment. Data are shown as mean±standard error (n = 5). In each row, *P*-values of Student’s *t*-test (U^-^ *vs* U^+^) are shown for each week of treatment (***P ≤ 0.001, **P ≤ 0.01, ns *P* > 0.05).

### 3.4 Levels of oxidative stress markers and hormones at post-inoculation stage

#### 3.4.1 Hydrogen peroxide content and lipid peroxidation

**Figure 4:**
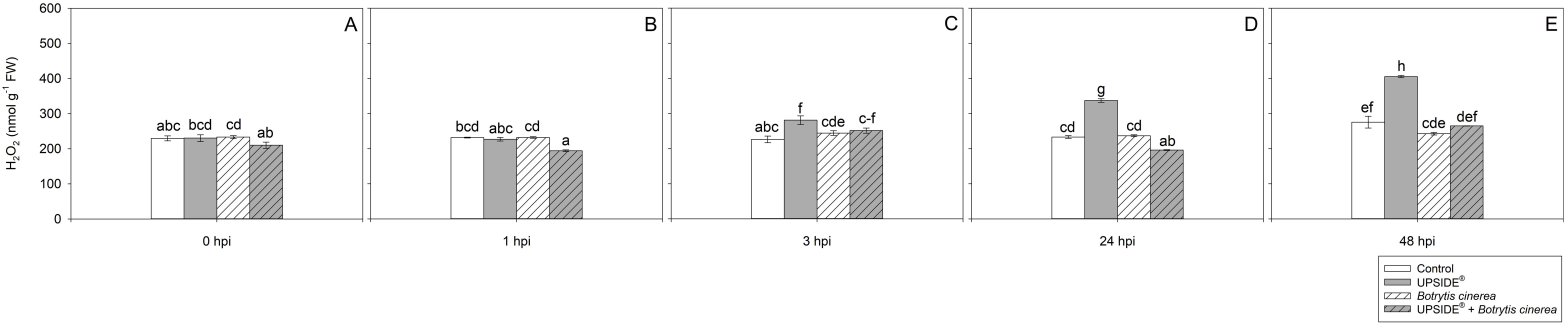
Variation in hydrogen peroxide (H_2_O_2_) content in *Vitis vinifera* cv. Sangiovese leaves treated with water (control, white bar), UPSIDE® (grey bar), inoculated with *Botrytis cinerea* (white filled bar), treated with UPSIDE® and inoculated with *B. cinerea* (grey filled bar). At least five plants were sampled at 0- **(A)**, 1- **(B)**, 3- **(C)**, 24- **(D)** and 48 **(E)** hours post inoculum (hpi). Data are shown as mean ± standard error (n = 5). In each graph, different letters indicate significant differences among means according to Tukey’s HSD *post hoc* test. FW, fresh weight.

The effect of ‘treatment’, ‘time’ and their interaction was significant for H_2_O_2_ and MDA contents (**Table 2**). At 0 hpi, H_2_O_2_ levels did not statistically change among the different plant groups (**Figure 4A**). At 1 hpi, only H_2_O_2_ levels of U^+^/Bc^+^ plants were lower than U^-^/Bc^-^ and U^-^/Bc^+^ (–16% on average; **Figure 4B**). At 3 hpi, a slight increase in U^+^/Bc^-^ plants was observed in comparison to U^-^/Bc^-^ and U^-^/Bc^+^ ones (+24 and +12%; **Figure 4C**). At 24 hpi, H_2_O_2_ reached higher values in U^+^/Bc^-^ plants (+45%, in comparison to U^-^/Bc^-^ and U^-^/Bc^+^, and +72% in comparison to U^+^/Bc^+^), while lower levels in U^+^/Bc^+^ (–16%, averagely, in comparison to both U^-^/Bc^-^ and U^-^/Bc^+^, and –42% in comparison to U^+^/Bc^-^; **Figure 4D**). At 48 hpi, the trend was maintained for U^+^/Bc^-^, which increased over time in comparison to 0 hpi (+24, +49 and +79%, respectively) and reached the peak and maximum values recorded throughout the experiment (+56% on average, in comparison to U^-^/Bc^-^, U^-^/Bc^+^ and U^+^/Bc^+^; **Figure 4E**). Not other intense changes were observed over time, but minor statistical variations were observed at 48 hpi in U^-^/Bc^-^ in comparison to 0 hpi (+19%; **Figure 4E**), and at 3 and 48 hpi in U^+^/Bc^+^, in comparison to 0 hpi (+6 and +12%; **Figures 4C, E**).

**Table 2:** *P* levels (*** *P* ≤ 0.001, ** *P* ≤ 0.01, * *P* ≤ 0.05) of the two-way analysis of variance for the effects of ‘treatment’, ‘time’ and their interaction ‘treatment × time’ on hydrogen peroxide (H_2_O_2_), malondialdehyde (MDA), ethylene/jamonic acid (Et/JA) ratio, salicylic acid (SA) and abscisic acid (ABA) in Vitis vinifera cv. Sangiovese leaves treated with water or UPSIDE^®^, inoculated with *Botrytis cinerea* or treated with UPSIDE^®^ and inoculated with *B. cinerea*.

Parameter	Treatment	Time	Treatment × Time
H_2_O_2_ (nmol g^-1^ FW)	***	***	***
MDA (nmol g^-1^ FW)	***	***	***
Et/JA (nl µg^-1^ h^-1^ FW)	***	***	***
SA (µg g^-1^ FW)	**	***	**
ABA (ng g^-1^ FW)	*	***	***

**Figure 5:**
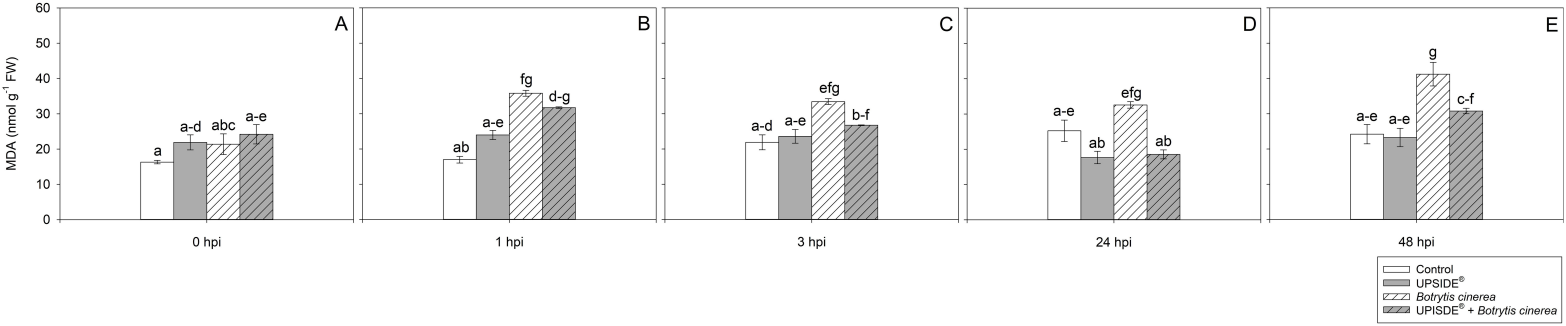
Variation in malondialdehyde (MDA) content in *Vitis vinifera* cv. Sangiovese leaves treated with water (control, white bar), UPSIDE® (grey bar), inoculated with *Botrytis cinerea* (white filled bar), treated with UPSIDE® and inoculated with *B. cinerea* (grey filled bar). At least five plants were sampled at 0- **(A)**, 1- **(B)**, 3- **(C)**, 24- **(D)** and 48 **(E)** hours post inoculum (hpi). Data are shown as mean ± standard error (n = 5). In each graph, different letters indicate significant differences among means according to Tukey’s HSD *post hoc* test. FW, fresh weight; hpi, hours post inoculum.

At 0 hpi, MDA values did not statistically change among the different plant groups (**Figure 5A**). At 1 hpi, MDA content increased in U^-^/Bc^+^ and U^+^/Bc^+^ in comparison to U^-^/Bc^-^ ones (more than 2-fold and +87%, respectively; **Figure 5B**). At 3 hpi, MDA levels increased only in U^-^/Bc^+^ plants (+53%, in comparison to U^-^/Bc^-^ ones; **Figure 5C**). Similarly, the content of MDA was higher in U^-^/Bc^+^ leaves in comparison to U^+^/Bc^-^ and U^+^/Bc^+^ ones (+80%, as average) at 24 hpi, and in comparison to all other groups at 48 hpi (+61%, averagely; **Figures 5D, E**). Significant differences over time were observed in U^-^/Bc^+^ plants throughout the experiment if compared to the results observed at 0 hpi (+51%, averagely; **Figure 5**). Overall, the results regarding oxidative stress indicated that U, alone, led to the increase of H_2_O_2_ values over time and in comparison to controls, while in U^+^/Bc^+^ the values tended to be lower at certain times (1 and 24 hpi); MDA increased from 1 hpi in U^-^/Bc^+^, until the end of the experiment, while in U^+^/Bc^+^ it increased only at 1 hpi.

#### 3.4.2 Hormones content

**Figure 6:**
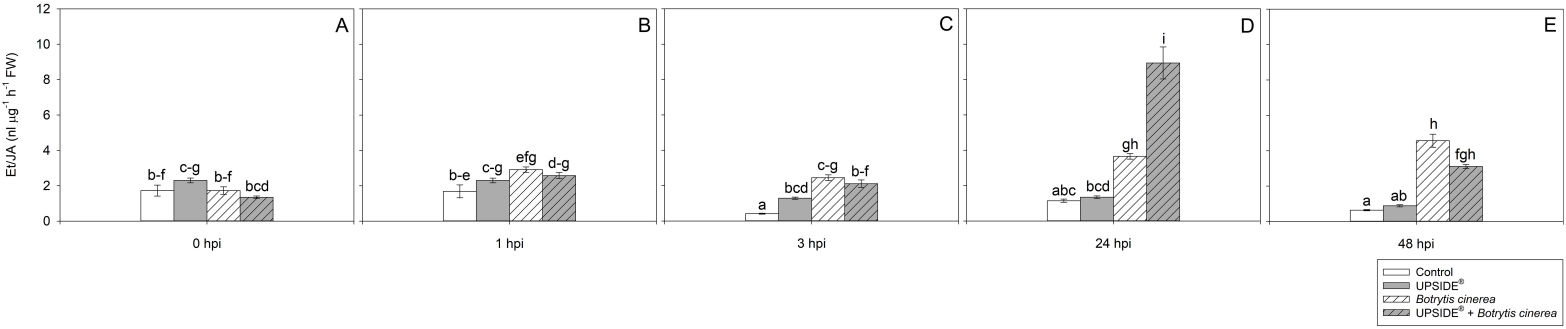
Variation in ethylene/jasmonic acid (Et/JA) ratio in *Vitis vinifera* cv. Sangiovese leaves treated with water (control, white bar), UPSIDE® (grey bar), inoculated with *Botrytis cinerea* (white filled bar), treated with UPSIDE® and inoculated with *B. cinerea* (grey filled bar). At least five plants were sampled at 0- **(A)**, 1- **(B)**, 3- **(C)**, 24- **(D)** and 48 **(E)** hours post inoculum (hpi). Data are shown as mean ± standard error (n = 5). In each graph, different letters indicate significant differences among means according to Tukey’s HSD *post hoc* test. FW, fresh weight; hpi, hours post inoculum.

The effect of ‘treatment’, ‘time’ and their interaction was significant for all the examined hormones (**Table 2**). No changes were observed at either 0 or 1 hpi (**Figures 6A, B**). However, at 3 hpi, the Et/JA ratio was significantly higher in U^+^/Bc^-^, and even more in both Bc^+^ plants (more than 3- and 5-fold, on average, in comparison to U^-^/Bc^-^ ones; **Figure 6C**). At 24 hpi, Et/JA reached a peak in U^-^/Bc^+^ and U^+^/Bc^+^ plants (more than 3- and 7-fold than U^-^/Bc^-^ ones; **Figure 6D**). Similarly, inoculated plants, regardless of the U treatment, showed an increase of Et/JA ratio also at 48 hpi (more than 7- and 5-fold, respectively for U^-^/Bc^+^ and U^+^/Bc^+^, in comparison to uninoculated plants; **Figure 6E**). Significant changes over time were observed in the different plant groups, in comparison to 0 hpi. The Et/JA ratio decreased in U^-^/Bc^-^ at both 3 and 48 hpi (–76 and –63%; **Figures 6C, E**), and in U^+^/Bc^-^ at 48 hpi (–62%; **Figure 6E**). Conversely, the values increased in U^-^/Bc^+^ and U^+^/Bc^+^ at 24 and 48 hpi (2- and more than 2-fold in U^-^/Bc^+^, more than 6- and more than 2-fold in U^+^/Bc^+^, respectively; **Figures 6D, E**).

**Figure 7:**
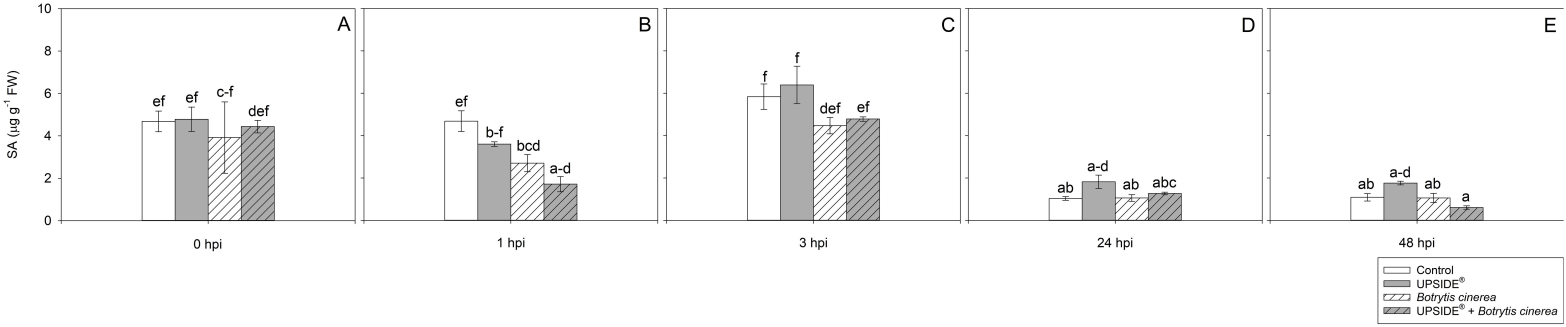
Variation in salicylic acid (SA) content in *Vitis vinifera* cv. Sangiovese leaves treated with water (control, white bar), UPSIDE® (grey bar), inoculated with *Botrytis cinerea* (white filled bar), treated with UPSIDE® and inoculated with *B. cinerea* (grey filled bar). At least five plants were sampled at 0- **(A)**, 1- **(B)**, 3- **(C)**, 24- **(D)** and 48 **(E)** hours post inoculum (hpi). Data are shown as mean ± standard error (n = 5). In each graph, different letters indicate significant differences among means according to Tukey’s HSD *post hoc* test. FW, fresh weight; hpi, hours post inoculum.

**Figure 8:**
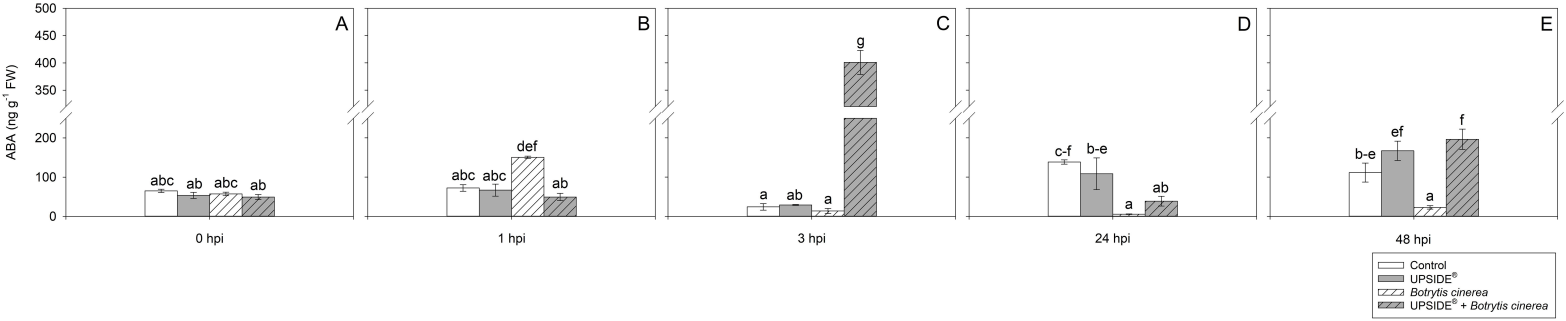
Variation in abscisic acid (ABA) content in *Vitis vinifera* cv. Sangiovese leaves treated with water (control, white bar), UPSIDE® (grey bar), inoculated with *Botrytis cinerea* (white filled bar), treated with UPSIDE® and inoculated with *B. cinerea* (grey filled bar). At least five plants were sampled at 0- **(A)**, 1- **(B)**, 3- **(C)**, 24- **(D)** and 48 **(E)** hours post inoculum (hpi). Data are shown as mean ± standard error (n = 5). In each graph, different letters indicate significant differences among means according to Tukey’s HSD *post hoc* test. FW, fresh weight; hpi, hours post inoculum.

At 0 hpi, all plant groups showed similar SA levels (**Figure 7A**). At 1 hpi, SA levels were lower in U^-^/Bc^+^ and in U^+^/Bc^+^ in comparison to U^-^/Bc^-^ plants (–42 and –63%, respectively; **Figure 7B**). No other changes were observed due to inoculation or treatment at separate analysis time (see **Figure 7C**), while minor variations were observed throughout the experiment and in comparison to 0 hpi. The SA levels decreased at 24 and 48 hpi in U^-^/Bc^-^ (-80%, as average, in comparison to all other time; **Figures 7D, E**). A decrease of SA was observed also in U^+^/Bc^-^, U^-^/Bc^+^, and U^+^/B^+^ at 24 and 48 hpi in comparison to the level recorded at 0 hpi (–60, –73 and –80%, as average). Abscisic acid content did not change at 0 hpi (**Figure 8A**). It notably increased at 1 hpi only in U^-^/Bc^+^ (more than 2-fold, on average, in comparison to the other treatment combinations; **Figure 8B**). A great peak was observed in U^+^/Bc^+^ at 3 hpi (more than 19-fold higher than U^-^/Bc^-^, U^+^/Bc^-^ and U^-^/Bc^+^; **Figure 8C**). At 24 hpi, ABA decreased in U^-^/Bc^+^ (–95%, averagely, in comparison to uninoculated plants) and in U^+^/Bc^+^ (–72% than U^-^/Bc^-^; **Figure 8D**). At 48 hpi, ABA content in U^+^/Bc^-^ and U^+^/Bc^+^ plants resulted higher than U^-^/Bc^+^ ones (8-fold higher, on average; **Figure 8E**). Over time, ABA never changed in controls, but it increased in comparison to 0 hpi in U^+^/Bc^-^ at 48 hpi (3-fold; **Figure 8E**), in U^-^/Bc^+^ at 1 hpi (more than 2-fold) and in U^+^/Bc^+^ at both 3 and 48 hpi (more than 8-fold and 4-fold higher, respectively; **Figures 8B, C, E**). No significant differences were recorded in the remaining sampling times.

In summary, data regarding hormones highlighted a small increase in the Et/JA ratio at 3 hpi in all plant groups, but the most relevant variations were observed in U^-^/Bc^+^ and U^+^/Bc^+^ at 24 and 48 hpi; no particular peaks were observed in SA content, which, by contrast decreased over time; ABA content increased firstly in U^-^/Bc^+^ plant (1 hpi) and, subsequently, in U^+^/Bc^+^ a great peak was detected at 3 hpi.

### 3.5 Canonical discriminant analysis and Pearson’s correlation

**Figure 9:**
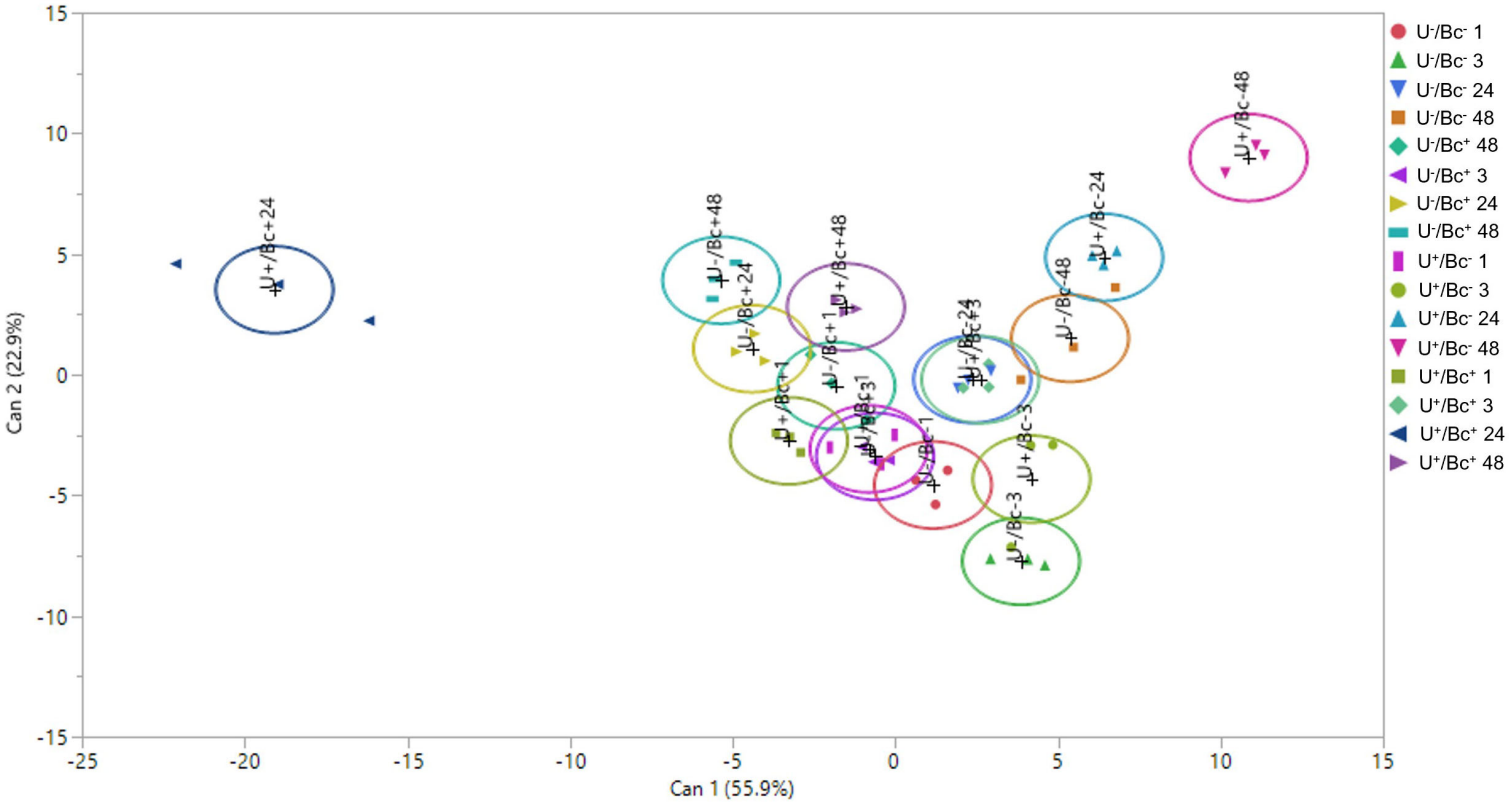
Two-dimensional scatterplot related to the Canonical Discriminant Analysis (CDA) performed on individual replicates, showing the separation of experimental groups based on hormones analyzed (Et/JA, SA, ABA and H_2_O_2_). Can 1, canonical function 1; Can 2, canonical function 2.

A CDA was performed to evaluate whether, and how, the measured variables differentially influenced the different groups across different sampling times and to assess the extent to which these variables aligned with hormones analyzed (Et/JA, SA, ABA and H_2_O_2_; **Figure 9**). While the overall trend of the CDA revealed a limited segregation among the majority of groups, a distinct separation within the multivariate hyperspace was observed between U^+^/Bc^+^ at 24 hpi and U^+^/Bc^-^ at 48 hpi, and between U^+^/Bc^+^ at 24 hpi and other groups, included U^+^/Bc^-^ at 24 hpi. These clusters were primarily associated with Bc infection, regardless of the U treatment and, secondarily, the time of analysis. This differentiation was outlined by Can 1, which explained 55.9% of the total variation. By contrast, based on Can 2, on the y-axis of the plot, which explained 22.9% of the total variation, the main clusters are correlated with the application of U, regardless of the fungal inoculation and time of analysis, as demonstrated by the hyperspace distance detected between U^-^/Bc^-^ at 3 hpi and U^+^/Bc^-^ at 48 hpi. Moreover, Pearson’s correlation coefficients based on the CDA scores showed a strong positive correlation between the Et/JA ratio and Can 1, as between SA and Can 2, and between H_2_O_2_ and both Can 1 and Can 2 (**Table 3**).

**Table 3:** Pearson’s correlation coefficients (r) between individual variables (hormones, i.e. Et/JA, SA, ABA and H_2_O_2_) and the first canonical function scores from the Canonical Discriminant Analysis (CDA).

	Pearson coefficient (|r|)	
	Can 1	Can 2
Et/JA	0.94***	0.20
SA	0.27	0.71**
ABA	0.23	0.30
H_2_O_2_	0.69*	0.61*

*0.5 > |r| > 0.7; **0.7 > |r| > 0.9; ***0.9 > |r| > 1.

### 3.6 Microscopic observations

**Figure 10:**
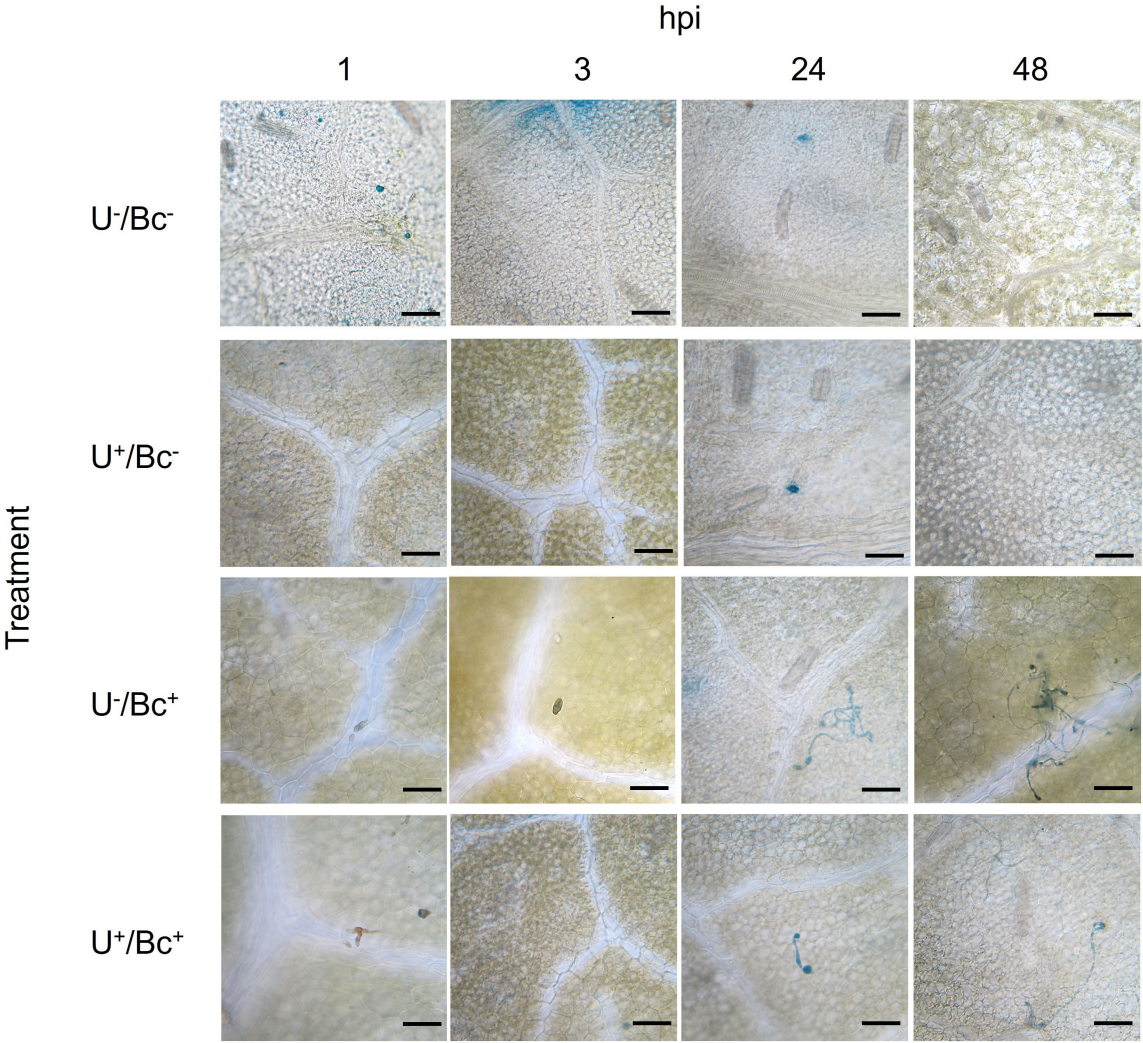
Leaves of *Vitis vinifera* cv. Sangiovese colored with lactophenol-cotton blue and observed in an optical at 1, 3, 24 and 48 hours post inoculation (hpi) of each treatment. From up to bottom: leaves treated with water (U^-^/Bc^-^), with UPSIDE^®^ (U^+^/Bc^-^), inoculated with *Botrytis cinerea* (U^-^/Bc^+^), treated with UPSIDE^®^ and inoculated with *B. cinerea* (U^+^/Bc^+^). Bar = 50 µm.

Microscopic observations allowed a first evaluation of the effective penetration of Bc in different areas of the leaves. In U^-^/Bc^-^ and U^+^/Bc^-^ plants, any stained fungal structure of Bc was observed, as expected (**Figure 10**). In U^-^/Bc^+^ plants, germ tubes emerged from conidia and elongated starting from 24 hpi. Their hyphae spread throughout leaf tissues until the end of the experiment. Even if similar structures were found in U^+^/Bc^+^, germ tubes resulted slightly formed and hyphae marginally spread over the leaves. The observed outcomes suggest an effect due to the U application.

## 4 Discussion

Defense priming is widely recognized as a highly cost-effective and efficient strategy of IR, enabling plants to conserve metabolic resources by avoiding unnecessary defense activation. Unlike constitutively expressed defenses, priming is typically associated with reduced fitness costs, supporting its role as an adaptive ecological mechanism that facilitates rapid and efficient responses to environmental stressors (Devi et al., 2023). However, elucidating the exact mechanism of action of a IR-inducer remains challenging, as it requires multiple analyses that are often labor-intensive and time-consuming. In Scimone et al. (2024), we conducted a greenhouse experiment to uncover the specific defense signals activated by U (so far named “yeast extract-based product”) in healthy grapevines both at biochemical and molecular levels. Our findings revealed a triggered JA-ABA mediated response and the up regulation of specific genes (*chit1b*, *pr2*, *hsr1*). Building on this previous work, the present study aimed to assess the efficacy of the yeast-inert-fraction based product as defense priming against Bc, and investigating again plant responses both prior to inoculation, and during the early stages following Bc inoculation, to better understand the dynamics of priming under biotic stress.

The first question to answer was: “Does U trigger biochemical defense responses in uninoculated grapevines at foliar level?”. In this study, U application in non-inoculated plants clearly induced a reduction of SA content at 24 h post treatment during the second week of experiment, an increase of H_2_O_2_ levels throughout the experiment, and a concomitant rise of Et/JA ratio. These results are supported by our previous findings (Scimone et al., 2024) and confirm the absence of SA involvement among the defense pathways. The decrease of this signaling molecule together with the accumulation of H_2_O_2_ suggests its crucial role as a regulator of cell survival to counteract ROS accumulation through the activation of phenylpropanoids pathway (Pellegrini et al., 2016; Ali, 2021; Dumanović et al., 2021). Concomitantly, the overall increase of H_2_O_2_ should not be considered as the consequence of a stressful event, but mostly as an activator of signaling cascade in plant defensive pathway (Černý et al., 2018; Smirnoff and Arnaud, 2019). Indeed, even if its presence is remarkably high throughout the U application time, unchanged MDA values (parameter always associated with lipid peroxidation due to oxidative stress; Toral et al., 2020) were observed at the end of the three weeks treatment, thus reflecting any detrimental effect due to the product application. A similar trend for H_2_O_2_ was found in U^+^/Bc^-^ plants included among the post inoculation set, by showing an increase of this signaling molecule starting from 3 hpi until the end of the experiment. This further highlights the pivotal role of this molecule, whose effect endures due to its accumulation and the resulting signaling activity even 24 h after the final recommended treatment. Notably, the accumulation of H_2_O_2_ underscores its function as a key of mediator cell-to-cell signaling during plant priming and the enhancement of defense responses following the application of U. Again, this occurs without inducing phytotoxic effects or membrane damage, as evidenced by unchanged MDA levels. In a similar manner to Nie et al. (2017), who investigated the use of *Bacillus cereus* AR156 as an inducer of induced systemic resistance in *Arabidopsis thaliana* against *Pseudomonas syringae*, the role of H_2_O_2_ was emphasized as a key activator of the downstream signaling cascade. This activation leads to the engagement of both Et and JA pathways, often represented as a ratio due to their high level of interconnection, as Et can stimulate JA biosynthesis and viceversa. These pathways are particularly important for resistance against necrotrophic pathogens (Xue et al., 2021; Mazuecos-Aguilera et al., 2025). Here, the present but transient activation of the Et/JA ratio observed at 3 hpi in U^+^/Bc^-^ plants indicates these hormones may be involved at early stages in U-priming-induced signaling responses. Therefore, the specific contribution of Et alone should be assessed at earlier time points following treatment (i.e., within the first two minutes; *data not shown*). In this context, it can be stated that U^+^/Bc^-^ plants acquired a form of preventive ‘training’. The activation of ABA at different time points supports the ability of the treatment to induce a primed physiological state. This enhanced status enables U-treated plants to more effectively allocate resources when faced with biotic challenges (Vos et al., 2015; Shigenaga et al., 2017; Puccioni et al., 2025).

Consequently, the second question was: “Does the signal similarly occur also in inoculated ones?”. The presence of Bc remarkably stimulated the Et/JA ratio throughout the experiment in U^-^/Bc^+^ plants, while a decrease of SA was observed at 1 hpi. These results are consistent with existing literature, which identifies the Et/JA-mediated pathway as a key player responsible for the modulation of the necrotrophic infection at an early stage, while repressing SA signal (as those molecules usually act antagonistically; Li et al., 2019; De Bona et al., 2021; Wang et al., 2021). Moreover, the increase in ABA content at 1 hpi suggests an initial attempt by the plant to avoid Bc passive penetration, as the presence of this phytohormone is frequently linked to stomatal closure, which can be determinant to prevent pathogen invasion during the first stages of infection (Du et al., 2024). However, this necrotrophic fungus also owns the ability to actively infect and colonize the tissue through appressorium-like structures which produce phytotoxins and cell-wall-degrading enzymes, while interfering with the host metabolism and determining ROS accumulation *in planta* (Haile et al., 2017; Liu et al., 2019; Modesti et al., 2023). Increased MDA content was recorded throughout the experiment, confirming the infectious process was onsite (Cela et al., 2018). Similar trends were observed in U^+^/Bc^+^ plants, where elevated Et/JA ratio and reduced SA levels were also observed. It is worth noting that the timing of signal activation appears crucial to repress a specific infectious disease. Near to this, the great peak of Et/JA in U^+^/Bc^+^ at 24 hpi, preceded by Et/JA-ABA interplay, may have a role in the reinforcement of chemical and physical barriers, which resulted in reduced fungal development one day after the inoculation (as confirmed by microscopic observations). Overall, it is also important to note that the different variations observed over the course of the experimental time points may be influenced by two main factors. The first is the circadian rhythm, which is known to regulate plant susceptibility to numerous pathogens and insects and has been extensively studied in model plants (e.g., *Arabidopsis thaliana*) and modulates the defense-related hormones as JA and SA (Venkat and Muneer, 2022). The second factor is the fungal development, which in turn can be influenced by its circadian clock or the application of a treatment, consequently triggering plant responses, either early or delayed (Hevia et al., 2015; Ingle et al., 2015). At 48 hpi, once the apparent risk of infection seemed reduced and conidial germination was modified, the signal appeared not so evident as in emergency-stage. To evidence that, precisely at 24 hours, the group in question is clearly distant both from most of the groups and especially from the clusters that, within the same time range, were only inoculated (and not U-treated). Conversely, when U is not applied, the crosstalk between the different hormones, later activated, did not stop the Bc growth, thus limiting the success of an effective plant resistance. The increased but transient ABA levels were also observed in our previous work (Scimone et al., 2024) and further confirm this phytohormone-related priming effect due to product application. Additionally, at the end of the trial unchanged MDA values were observed, thus suggesting the mitigatory activity of U in alleviating Bc induced damages. Similar outcomes resulted from the application of other biocontrol agents such as *B. velezensis*, *Streptomyces pratensis*, *Trichoderma harzianum* in strawberry and tomato, respectively (Lian et al., 2017; Toral et al., 2020; Geng et al., 2022).

Lastly, the third question aimed to answer was: “Does U have any fungistatic/fungicidal effects on Bc?”. Here, the observations made through *in vitro* test confirmed the direct inhibition that U determined on Bc-mycelium growth. Similarly, at the optical microscope it was revealed Bc grew during the time over the U^-^/Bc^+^ leaves surface, while the fungus hyphae presumably stopped growing, due to their misshaped appearance, on the U^+^/Bc^+^ ones. It is well documented living yeasts application can directly affect Bc mycelium growth, through the production of killer toxins or cell wall-degrading enzymes among with volatile organic compounds emission (Santos et al., 2004; Lopes et al., 2015; Maluleke et al., 2022). However, few have been recorded for yeast-inert-fraction based products so far. According to Sun et al. (2018), the presence of chitin, a polysaccharide polymer with an important structural role within the yeast cell wall, could play a key role in terms of antifungal activity as its application was proved to be efficient against Bc on tomato fruits. Moreover, in De Bona et al. (2021) work, chitosan, a chitin derivate, manifested a direct fungistatic effect against Bc inoculated on amended PDA. In addition, chitosan already showed strong capability to control Bc germination, in a dose-dependent manner, disrupting fungal cell membrane (by affecting the electrostatic balance of phospholipids), and, consequently, inhibiting nucleic acid synthesis (Reglinski et al., 2010; Kim et al., 2019). Thus, U, not only determines the activation of defense system at leaf level but also owns antifungal properties due to its composition. Consequently, this highlights its dual mode of action against Bc: an indirect action due to the induction of plant resistance and a direct fungistatic effect.

This raises an additional question regarding the specific focus of our work: “Is the reduction in Bc mycelial growth induced by U primarily due to its antifungal activity or its IR-inducing effect?”

Based on the tests performed, it is not possible to attribute the observed reduction in Bc to a single mode of action of U.

Although similar products have been extensively studied by other researchers, this aspect has not been directly addressed. Evidence indicates that chitosan application promotes the accumulation of secondary metabolites—such as stilbene phytoalexins (*trans*- and *cis*-resveratrol, and *ϵ*-viniferins)—and enhances chitinase and β-1,3-glucanase activities. These compounds exhibit broad-spectrum antimicrobial activity at physiological concentrations by activating resistance to Bc and thereby reducing mycelial development in grapevine leaves. However, the study by Aziz et al. (2006) suggested that the overall reduction in Bc growth was not directly caused by the active compound’s inhibitory effect on the pathogen itself.

In our study, given the demonstrated dual mode of action of U, the observed decrease in Bc mycelial growth on leaf surfaces likely results from the additive interaction between its direct antifungal and indirect IR-inducing effects. Further targeted research is needed to clarify and quantify the relative contributions of each mechanism to the overall protective response.

The outcomes of this study represent a forward step in the field of vineyard disease management, as U can satisfy the demand for new alternative products to use against Bc. Indeed, as reported from the last research trends highlighted by selected keywords on Scopus, there is consistent and growing interest in developing new sustainable alternatives to exploit in Bc management. The clusters generated from the 90 documents reported show that, in 20 years, the interest shifted from studies focused merely on the grapevine and Bc itself, to applicative works which rely on applied prevention and control approaches using BCAs.

In conclusion, the efficacy of U against grey mold was proved. Biochemical analyses showed that the tested formulate can stimulate increasing levels of key hormones implied in the defense system pathways such as Et/JA, ABA and H_2_O_2_, at early stages, both alone and within the presence of biotic challengers such as Bc (preventive and curative effect). Thus, confirming the ability of the product to induce IR as mode of action. Moreover, the product determined changes in the fungus hyphal structure, suggesting its efficacy also in terms of fungistatic effect. This is a pivotal goal in the development of emerging new techniques and novel methods to control grapevine diseases, by offering an alternative to the use of traditional chemicals in viticulture. Nonetheless, additional research is necessary to comprehensively assess the efficacy of U across another grapevine related pathosystems, and to elucidate the involvement of the signaling molecules at the biochemical and molecular level.

## Data Availability

The raw data supporting the conclusions of this article will be made available by the authors, without undue reservation.

## References

[B1] AbuQamar S. Moustafa K. Tran L. S. (2017). Mechanisms and strategies of plant defense against *Botrytis cinerea* . Crit. Rev. Biotechnol. 37, 262–274. doi: 10.1080/07388551.2016.1271767, PMID: 28056558

[B2] Ali B. (2021). Salicylic acid: an efficient elicitor of secondary metabolite production in plants. Biocatal. Agric. Biotechnol. 31, 101884. doi: 10.1016/j.bcab.2020.101884

[B3] Altieri V. Rossi V. Fedele G. (2024). Integration of mathematical modeling and target-based application of biocontrol agents for the control of *Botrytis cinerea* in vineyards. Pest Manage. Sci. 80, 4352–4360. doi: 10.1002/ps.8140, PMID: 38634563

[B4] Aziz A. Trotel-Aziz P. Dhuicq L. Jeandet P. Couderchet M. Vernet G. (2006). Chitosan oligomers and copper sulfate induce grapevine defense reactions and resistance to gray mold and downy mildew. Phytopathol 96, 1188–1194. doi: 10.1094/PHYTO-96-1188, PMID: 18943955

[B5] Brulé D. Héloir M. C. Roudaire T. Darblade B. Hugueney P. Poinssot B. (2024). Increasing vineyard sustainability: innovating a targeted chitosan-derived biocontrol solution to induce grapevine resistance against downy and powdery mildews. Front. Plant Sci. 15. doi: 10.3389/fpls.2024.1360254, PMID: 38384763 PMC10879612

[B6] Calvo-Garrido C. Viñas I. Elmer P. A. Usall J. Teixidó N. (2014). Suppression of *Botrytis cinerea* on necrotic grapevine tissues by early-season applications of natural products and biological control agents. Pest Manage. Sci. 70, 595–602. doi: 10.1002/ps.3587, PMID: 23744713

[B7] Carisse O. van der Heyden H. (2025). Knowledge-based integrated management of *Botrytis* bunch rot of grapevine caused by *Botrytis cinerea* in a northern climate. PhytoFront 10, 40. doi: 10.1094/PHYTOFR-12-24-0139-R

[B8] Cela J. Tweed J. K. Sivakumaran A. Lee M. R. Mur L. A. Munné-Bosch S. (2018). An altered tocopherol composition in chloroplasts reduces plant resistance to *Botrytis cinerea* . Plant Physiol. Biochem. 127, 200–210. doi: 10.1016/j.plaphy.2018.03.033, PMID: 29609176

[B9] Černý M. Habanova H. Berka M. Luklova M. Brzobohatý B. (2018). Hydrogen peroxide: its role in plant biology and crosstalk with signaling networks. Int. J. Mol. Sci. 19, 2812. doi: 10.3390/ijms19092812, PMID: 30231521 PMC6163176

[B10] Collinge D. B. Jensen D. F. Rabiey M. Sarrocco S. Shaw M. W. Shaw R. H. (2022). Biological control of plant diseases–What has been achieved and what is the direction? Plant Pathol. 71, 1024–1047. doi: 10.1111/ppa.13555

[B11] De Bona G. S. Vincenzi S. De Marchi F. Angelini E. Bertazzon N. (2021). Chitosan induces delayed grapevine defense mechanisms and protects grapevine against *Botrytis cinerea* . J. Plant Dis. Protect 128, 715–724. doi: 10.1007/s41348-021-00432-3

[B12] De Kesel J. Conrath U. Flors V. Luna E. Mageroy M. H. Mauch-Mani B.. (2021). The induced resistance lexicon: do’s and don’ts. Trends Plant Sci. 26, 685–691. doi: 10.1016/j.tplants.2021.01.001, PMID: 33531282

[B13] Devi B. Tiwari M. Yadav N. Singh P. (2023). Intergenerational immune priming: harnessing plant growth promoting rhizobacteria (PGPR) for augmented wheat protection against spot blotch. Physiol. Mol. Plant Pathol. 128, 102164. doi: 10.1016/j.pmpp.2023.102164

[B14] Du Y. Zhang H. Jia K. Chu Z. Xu S. Tran L. S. P.. (2024). Role of abscisic acid-mediated stomatal closure in responses to pathogens in plants. Physiol. Plant 176, e14135. doi: 10.1111/ppl.14135

[B15] Dumanović J. Nepovimova E. Natić M. Kuča K. Jaćević V. (2021). The significance of reactive oxygen species and antioxidant defense system in plants: a concise overview. Front. Plant Sci. 11. doi: 10.3389/fpls.2020.552969, PMID: 33488637 PMC7815643

[B16] EPPO Bulletin (2014). Efﬁcacy evaluation of plant protection products Vol. 44 (European and Mediterranean Plant Protection Organization), 265–273.

[B17] FAO-OIV (2016). FAO-OIV Focus 2016. Table and dried grapes (Rome: Food and Agriculture Organization of the United Nations).

[B18] Fedele G. Brischetto C. Rossi V. (2020). Biocontrol of *Botrytis cinerea* on grape berries as influenced by temperature and humidity. Front. Plant Sci. 11. doi: 10.3389/fpls.2020.01232, PMID: 32922419 PMC7457006

[B19] Fedorina J. Tikhonova N. Ukhatova Y. Ivanov R. Khlestkina E. (2022). Grapevine gene systems for resistance to gray mold *Botrytis cinerea* and powdery mildew *Erysiphe necator* . Agronomy 12, 499. doi: 10.3390/agronomy12020499

[B20] Gambetta G. A. Herrera J. C. Dayer S. Feng Q. Hochberg U. Castellarin S. D. (2020). The physiology of drought stress in grapevine: towards an integrative definition of drought tolerance. J. Exp. Bot. 71, 4658–4676. doi: 10.1093/jxb/eraa245, PMID: 32433735 PMC7410189

[B21] Geng L. Fu Y. Peng X. Yang Z. Zhang M. Song Z.. (2022). Biocontrol potential of *Trichoderma harzianum* against *Botrytis cinerea* in tomato plants. Biol. Control 174, 105019. doi: 10.1016/j.biocontrol.2022.105019

[B22] González-Domínguez E. Fedele G. Caffi T. Delière L. Sauris P. Gramaje D.. (2019). A network meta-analysis provides new insight into fungicide scheduling for the control of *Botrytis cinerea* in vineyards. Pest. Manage. Sci. 75, 324–332. doi: 10.1002/ps.5116, PMID: 29885027

[B23] Hahn M. (2014). The rising threat of fungicide resistance in plant pathogenic fungi: *Botrytis* as a case study. J. Chem. Biol. 7, 133–141. doi: 10.1007/s12154-014-0113-1, PMID: 25320647 PMC4182335

[B24] Haile Z. M. Pilati S. Sonego P. Malacarne G. Vrhovsek U. Engelen K.. (2017). Molecular analysis of the early interaction between the grapevine flower and *Botrytis cinerea* reveals that prompt activation of specific host pathways leads to fungus quiescence. Plant Cell Environ. 40, 1409–1428. doi: 10.1094/PHYTO.2004.94.8.850, PMID: 28239986

[B25] He D. C. He M. H. Amalin D. M. Liu W. AlvIndia D. G. Xhan J. (2021). Biological control of plant diseases: an evolutionary and eco-economic consideration. Pathogens 10, 1311. doi: 10.3390/pathogens10101311, PMID: 34684260 PMC8541133

[B26] Hevia M. A. Canessa P. Müller-Esparza H. Larrondo L. F. (2015). A circadian oscillator in the fungus *Botrytis cinerea* regulates virulence when infecting *Arabidopsis thaliana* . Proc. Natl. Acad. Sci. 112, 8744–8749. doi: 10.1073/pnas.1508432112, PMID: 26124115 PMC4507220

[B27] Hodges D. M. DeLong J. M. Forney C. F. Prange R. K. (1999). Improving the thiobarbituric acid-reactive-substances assay for estimating lipid peroxidation in plant tissues containing anthocyanin and other interfering compounds. Planta 207, 604–611. doi: 10.1007/s004250050524 28456836

[B28] Ingle R. A. Stoker C. Stone W. Adams N. Smith R. Grant M.. (2015). Jasmonate signalling drives time‐of‐day differences in susceptibility of *Arabidopsis* to the fungal pathogen *Botrytis cinerea* . Plant J. 84, 937–948. doi: 10.1111/tpj.13050, PMID: 26466558 PMC4982060

[B29] Jelenić J. Ilić J. Ćosić J. Vrandečić K. Velki M. (2025). Growing our own poison–a vicious circle of more fungicides and more resistant *Botrytis cinerea* isolates. J. Plant Pathol. 107(1), 53–65. doi: 0.1007/s42161-023-01587-8

[B30] Kim Y. C. Hur J. Y. Park S. K. (2019). Biocontrol of *Botrytis cinerea* by chitin-based cultures of *Paenibacillus elgii* HOA73. Eur. J. Plant Pathol. 155, 253–263. doi: 10.1007/s10658-019-01768-1

[B31] La Torre A. Mandalà C. Pezza L. Caradonia F. Battaglia V. (2014). Evaluation of essential plant oils for the control of *Plasmopara viticola* . J. Essent. Oil Res. 26, 282–291. doi: 10.1080/10412905.2014.889049

[B32] Li N. Han X. Feng D. Yuan D. Huang L. J. (2019). Signaling crosstalk between salicylic acid and ethylene/jasmonate in plant defense: do we understand what they are whispering? Int. J. Mol. Sci. 20, 671. doi: 10.3390/ijms20030671, PMID: 30720746 PMC6387439

[B33] Li T. Zhou J. Li J. (2023). Combined effects of temperature and humidity on the interaction between tomato and *Botrytis cinerea* revealed by integration of histological characteristics and transcriptome sequencing. Hortic. Res. 10, uhac257. doi: 10.1093/hr/uhac257, PMID: 36778184 PMC9907048

[B34] Lian Q. Zhang J. Gan L. Ma Q. Zong Z. Wang Y. (2017). The biocontrol efficacy of *Streptomyces pratensis* LMM15 on *Botrytis cinerea* in tomato. BioMed. Res. Int. 2017, 9486794. doi: 10.1155/2017/9486794, PMID: 29318156 PMC5727823

[B35] Liu Y. Liu J. K. Li G. H. Zhang M. Z. Zhang Y. Y. Wang Y. Y.. (2019). A novel *Botrytis cinerea*‐specific gene BcHBF1 enhances virulence of the grey mould fungus via promoting host penetration and invasive hyphal development. Mol. Plant Pathol. 20, 731–747. doi: 10.1111/mpp.12788, PMID: 31008573 PMC6637910

[B36] Lopes M. R. Klein M. N. Ferraz L. P. da Silva A. C. Kupper K. C. (2015). *Saccharomyces cerevisiae*: a novel and efficient biological control agent for *Colletotrichum acutatum* during pre-harvest. Microbiol. Res. 175, 93–99. doi: 10.1016/j.micres.2015.04.003, PMID: 25960430

[B37] Maluleke E. Jolly N. P. Patterton H. G. Setati M. E. (2022). Antifungal activity of non-conventional yeasts against *Botrytis cinerea* and non-*Botrytis* grape bunch rot fungi. Front. Microbiol. 13. doi: 10.3389/fmicb.2022.986229, PMID: 36081805 PMC9445577

[B38] Mauch-Mani B. Baccelli I. Luna E. Flors V. (2017). Defense priming: an adaptive part of induced resistance. Annu. Rev. Plant Biol. 68, 485–512. doi: 10.1146/annurev-arplant-042916-041132, PMID: 28226238

[B39] Mazuecos-Aguilera I. Anta-Fernández F. Crespo-Barreiro A. Martínez-Quesada A. Lombana-Larrea L. González-Andrés F. (2025). Plant growth-promoting rhizobacteria enhanced induced systemic resistance of tomato against *Botrytis cinerea* phytopathogen. Front. Plant Sci. 16. doi: 10.3389/fpls.2025.1570986, PMID: 40303853 PMC12038444

[B40] McLaughlin M. S. Yurgel S. N. Abbasi P. A. Ali S. (2024). The effects of chemical fungicides and salicylic acid on the apple microbiome and fungal disease incidence under changing environmental conditions. Front. Microbiol. 15. doi: 10.3389/fmicb.2024.1342407, PMID: 38374916 PMC10875086

[B41] Modesti M. Marchica A. Pisuttu C. Risoli S. Pellegrini E. Bellincontro A.. (2023). Ozone-induced biochemical and molecular changes in *Vitis vinifera* leaves and responses to *Botrytis cinerea* infections. Antioxidants 12, 343. doi: 10.3390/antiox12020343, PMID: 36829902 PMC9952442

[B42] Mundy D. C. Elmer P. Wood P. Agnew R. (2022). A review of cultural practices for botrytis bunch rot management in New Zealand vineyards. Plants 11, 3004. doi: 10.3390/plants11213004, PMID: 36365455 PMC9657730

[B43] Nie P. Li X. Wang S. Guo J. Zhao H. Niu D. (2017). Induced systemic resistance against *Botrytis cinerea* by *Bacillus cereus* AR156 through a JA/ET-and NPR1-dependent signaling pathway and activates PAMP-triggered immunity in Arabidopsis. Front. Plant Sci. 8. doi: 10.3389/fpls.2017.00238, PMID: 28293243 PMC5329000

[B44] O’Brien P. A. (2017). Biological control of plant diseases. Australas Plant Pathol. 46, 293–304. doi: 10.1007/s13313-017-0481-4

[B45] Palmieri D. Ianiri G. Del Grosso C. Barone G. De Curtis F. Castoria R.. (2022). Advances and perspectives in the use of biocontrol agents against fungal plant diseases. Horticulturae 8, 577. doi: 10.3390/horticulturae8070577

[B46] Pellegrini E. Trivellini A. Cotrozzi L. Vernieri P. Nali C. (2016). “Involvement of phytohormones in plant responses to ozone,” in Plant hormones under challenging environmental factors. Eds. Ahammed G. Yu J. Q. (Springer, Dordrecht), 215–245.

[B47] Pertot I. Giovannini O. Benanchi M. Caffi T. Rossi V. Mugnai L. (2017). Combining biocontrol agents with different mechanisms of action in a strategy to control *Botrytis cinerea* on grapevine. Crop Prot 97, 85–93. doi: 10.1016/j.cropro.2017.01.010

[B48] Pisuttu C. Pellegrini E. Cotrozzi L. Nali C. Lorenzini G. (2020). Ecophysiological and biochemical events associated with the challenge of *Verticillium dahliae* to eggplant. Eur. J. Plant Pathol. 158, 879–894. doi: 10.1007/s10658-020-02122-6

[B49] Pisuttu C. Risoli S. Moncini L. Nali C. Pellegrini E. Sarrocco S. (2023). Sustainable strategies to counteract mycotoxins contamination and cowpea weevil in chickpea seeds during post-harvest. Toxins 15, 61. doi: 10.3390/toxins15010061, PMID: 36668881 PMC9865523

[B50] Provost C. Pedneault K. (2016). The organic vineyard as a balanced ecosystem: Improved organic grape management and impacts on wine quality. Sci. Hort 208, 43–56. doi: 10.1016/j.scienta.2016.04.024

[B51] Puccioni S. Biselli C. Perria R. Zanella G. D’Arcangelo M. E. M. (2025). Alternative effects yeast-based biostimulants against downy mildew in *Vitis vinifera* cv Cabernet Sauvignon. Hortic 11, 203. doi: 10.3390/horticulturae11020203

[B52] Rahman M. U. Liu X. Wang X. Fan B. (2024). Grapevine gray mold disease: infection, defense and management. Hortic. Res. 11, uhae182. doi: 10.1093/hr/uhae182, PMID: 39247883 PMC11374537

[B53] Raymaekers K. Ponet L. Holtappels D. Berckmans B. Cammue B. P. (2020). Screening for novel biocontrol agents applicable in plant disease management–a review. Biol. Control 144, 104240. doi: 10.1016/j.biocontrol.2020.104240

[B54] Reddy C. A. Oraon S. Bharti S. D. Yadav A. K. Hazarika S. (2024). Advancing disease management in agriculture: a review of plant pathology techniques. Plant Sci. Arch., 16–18. doi: 10.5147/PSA.2024.9.1.16

[B55] Reglinski T. Elmer P. A. G. Taylor J. T. Wood P. N. Hoyte S. M. (2010). Inhibition of *Botrytis cinerea* growth and suppression of botrytis bunch rot in grapes using chitosan. Plant Pathol. 59, 882–890. doi: 10.1111/j.1365-3059.2010.02312.x

[B56] Santin M. Caturegli L. Gagliardi L. Luglio S. M. Magni S. Pellegrini E.. (2025). Innovative techniques for managing dollar spot in warm-and cool-season turfgrasses: the case of UV-B and UV-C irradiations. Agriculture 15, 784. doi: 10.3390/agriculture15070784

[B57] Santos A. Sánchez A. Marquina D. (2004). Yeasts as biological agents to control *Botrytis cinerea* . Microbiol. Res. 159, 331–338. doi: 10.1016/j.micres.2004.07.001, PMID: 15646379

[B58] Scimone G. Lauria G. Pieracci Y. Cotrozzi L. Flamini G. Nali C.. (2025). Alternative approaches to enhance sweet basil (*Ocimum basilicum* L.) essential oil: improving composition, yield, and antifungal efficacy against *Botrytis cinerea* Pers. with monochromatic light and ozonated water. Ind. Crops Prod. 232, 121267. doi: 10.1016/j.indcrop.2025.121267

[B59] Scimone G. Vicente I. Bartalena G. Pisuttu C. Mariotti L. Risoli S.. (2024). Unravelling the biochemical and molecular priming effect of a new yeast-derived product: new perspectives towards disease management. Agriculture 14, 1047. doi: 10.3390/agriculture14071047

[B60] Shigenaga A. M. Berens M. L. Tsuda K. Argueso C. T. (2017). Towards engineering of hormonal crosstalk in plant immunity. Curr. Opin. Plant Biol. 38, 164–172. doi: 10.1016/j.pbi.2017.04.021, PMID: 28624670

[B61] Shu P. Li Y. Sheng J. Shen L. (2024). Recent advances in dissecting the function of ethylene in interaction between host and pathogen. J. Agric. Food Chem. 72, 4552–4563. doi: 10.1021/acs.jafc.3c07978, PMID: 38379128

[B62] Smirnoff N. Arnaud D. (2019). Hydrogen peroxide metabolism and functions in plants. New Phytol. 221, 1197–1214. doi: 10.1111/nph.15488, PMID: 30222198

[B63] Sun C. Fu D. Jin L. Chen M. Zheng X. Yu T. (2018). Chitin isolated from yeast cell wall induces the resistance of tomato fruit to *Botrytis cinerea* . Carbohydr. Polym. 199, 341–352. doi: 10.1016/j.carbpol.2018.07.045, PMID: 30143138

[B64] Thambugala K. M. Daranagama D. A. Phillips A. J. Kannangara S. D. Promputtha I. (2020). Fungi vs. fungi in biocontrol: an overview of fungal antagonists applied against fungal plant pathogens. Front. Cell. Infect. Microbiol. 10. doi: 10.3389/fcimb.2020.604923, PMID: 33330142 PMC7734056

[B65] Toffolatti S. L. Maddalena G. Marcianò D. Passera A. Quaglino F. (2020). A molecular epidemiology study reveals the presence of identical genotypes on grapevines and ground cover weeds and the existence of separate genetic groups in a *Botrytis cinerea* population. Plant Pathol. 69, 1695–1707. doi: 10.1111/ppa.13257

[B66] Toral L. Rodríguez M. Béjar V. Sampedro I. (2020). Crop protection against *Botrytis cinerea* by rhizhosphere biological control agent *Bacillus velezensis* XT1. Microorganisms 8, 992. doi: 10.3390/microorganisms8070992, PMID: 32635146 PMC7409083

[B67] Urban L. Lauri F. Ben Hdech D. Aarrouf J. (2022). Prospects for increasing the efficacy of plant resistance inducers stimulating salicylic acid. Agronomy 12, 3151. doi: 10.3390/agronomy12123151

[B68] van Hulten M. Pelser M. Van Loon L. C. Pieterse C. M. Ton J. (2006). Costs and benefits of priming for defense in *Arabidopsis* . Proc. Natl. Acad. Sci. 103, 5602–5607. doi: 10.1073/pnas.0510213103, PMID: 16565218 PMC1459400

[B69] Venkat A. Muneer S. (2022). Role of circadian rhythms in major plant metabolic and signaling pathways. Front. Plant Sci. 13. doi: 10.3389/fpls.2022.836244, PMID: 35463437 PMC9019581

[B70] Vos C. M. F. De Cremer K. Cammue B. P. A. De Coninck B. (2015). The toolbox of *Trichoderma* spp. in the biocontrol of *Botrytis cinerea* disease. Mol. Plant Pathol. 16, 400–412. doi: 10.1111/mpp.12189, PMID: 25171761 PMC6638538

[B71] Walker A. S. Ravigné V. Rieux A. Ali S. Carpentier F. Fournier E. (2017). Fungal adaptation to contemporary fungicide applications: the case of *Botrytis cinerea* populations from Champagne vineyards (France). Mol. Ecol. 26, 1919–1935. doi: 10.1111/mec.14072, PMID: 28231406

[B72] Wang R. Chen D. Khan R. A. A. Cui J. Hou. J. Liu T. (2021). A novel *Trichoderma asperellum* strain DQ-1 promotes tomato growth and induces resistance to gray mold caused by *Botrytis cinerea* . FEMS Microbiol. Lett. 368, fnab140. doi: 10.1093/femsle/fnab140, PMID: 34751779

[B73] Xue X. Geng T. Liu H. Yang W. Zhong W. Zhang Z.. (2021). Foliar application of silicon enhances resistance against *Phytophthora infestans* through the ET/JA-and NPR1-dependent signaling pathways in potato. Front. Plant Sci. 12. doi: 10.3389/fpls.2021.609870, PMID: 33584769 PMC7876464

